# Eating Motives and Other Factors Predicting Emotional Overeating during COVID-19 in a Sample of Polish Adults

**DOI:** 10.3390/nu13051658

**Published:** 2021-05-13

**Authors:** Adriana Modrzejewska, Kamila Czepczor-Bernat, Justyna Modrzejewska, Paweł Matusik

**Affiliations:** 1 Department of Psychology, Katowice Business University, 40-659 Katowice, Poland; 2 Institute of Psychology, University of Wroclaw, 50-527 Wroclaw, Poland; kamila.czepczor-bernat@uwr.edu.pl; 3 Institute of Pedagogy, University of Bielsko-Biala, 43-309 Bielsko-Biala, Poland; jmodrzejewska@ath.bielsko.pl; 4 Department of Pediatrics and Pediatric Endocrinology, Faculty of Medical Sciences in Katowice, Medical University of Silesia, 40-752 Katowice, Poland; pmatusik73@gmail.com

**Keywords:** eating motives, COVID-19-related stress, affect regulation, emotional overeating, COVID-19

## Abstract

We hypothesised that the higher levels of emotion-related predictors (eating motive in the form of affect regulation and COVID-19-related stress) would be associated with higher emotional overeating, after accounting for the effects of demographic variables (gender and BMI) and other eating motives (visual- and attitude-related predictors: liking, pleasure, visual appeal; body- and health-related predictors: need and hunger, health, weight control). Participants (*N* = 868; *M*_age_ = 33.53 years, *SD* = 11.98) completed: the Eating Motivation Survey, the Emotional Overeating Questionnaire, a COVID-19-related stress measure and a socio-demographic survey. The final step of the regression with emotional overeating was significant; affect regulation and COVID-19-related stress were significantly related to emotional overeating (Δ*F* *p* < 0.001, Adj. Δ*R*^2^ = 0.13). During the COVID-19 pandemic, eating can, on the one hand, help to cope with the current difficult situation and the negative emotions associated with it; on the other hand, frequent use of this tendency can lead to rigid regulation of affect and use of this mechanism as the dominant mechanism. Therefore, limited social contact, related disruptions in daily activities and stress resulting from COVID-19 should generate appropriate interventions, not necessarily focusing only on emotional eating, but also on the resources of the individual. It is worth encouraging specialists to implement alternative methods of contact with their patients, e.g., online.

## 1. Introduction

COVID-19 is a serious threat to people around the world [1,2]. In order to reduce the likelihood of contracting the coronavirus, many countries have introduced a lockdown, including, among other measures, a significant reduction in social contact and restrictions on travelling and using places where people meet and stay (including gyms, cinemas, shopping centres, universities) [1,2]. While these prevention measures may effectively protect people from being infected, they also cause significant changes in people’s behavioural and social functioning, which can contribute to deteriorating mental functioning [1,2,3,4,5,6,7]. The COVID-19-related situation is the cause of, among other things, concerns for the health of oneself and those of loved ones, as well as worries about the unstable economic situation and this, in turn, leads to an increase in the level of daily stress [5,8]. Limited social contact and another introduced restrictions make it much more difficult to deal with emotions and stress through adaptive mechanisms (e.g., through direct contact and conversation with another person, through regular physical activity, through contact with nature) and this fosters the development and/or strengthening of maladaptive coping strategies, one of which is emotional overeating [9,10,11,12,13,14].

The novel coronavirus pandemic has significantly disrupted people’s daily routines and lifestyles [15,16,17,18,19]. These changes concern, among others, eating behaviour, which is often less healthy and, along with decreased levels of physical activity, leads to significant weight gain [17,18,19,20]. It may result in long-term consequences of COVID-19 in the form of an increase in the prevalence of weight problems and obesity in the world [17,18,19,20]. This is especially dangerous as more and more reports say that obese people are at high risk of complications from COVID-19 [21,22,23]. It is therefore worth investigating eating motives and other factors predicting emotional overeating during COVID-19.

There is extensive literature on eating motives and other determinants of emotional overeating [24,25,26]. By asking the question, “Why do people eat what they eat?”, we can list many factors (e.g., physiological, economic, health, emotional, socio-environmental, attitudinal) [24]. Therefore, in the present study, only some of the variables were selected. In line with previous studies, it was assumed that significant predictors of emotional overeating would be [10,27,28,29,30,31,32,33,34]: (a) demographic predictors: female gender and body mass index; (b) visual- and attitude-related predictors: liking, pleasure, visual appeal; (c) body- and health-related predictors: need and hunger, health, weight control; (d) emotion-related predictors: affect regulation, COVID-19-related stress.

Visual- and attitude-related eating motives are associated with a positive attitude towards eating which is based on the visual qualities of food and the act of liking and enjoying eating [24]. Research shows that the relationship between these predictors, BMI and emotional overeating will be positive [10,27,29,30,31,32,33,35,36]. The next group of eating motives—body- and health-related factors—is related to: a physiological need for energy, the state of hunger vs. satiety and the motivation to be healthy and maintain a balanced diet and a healthy weight [24]. In turn, these predictors will be connected by a negative relationship with emotional overeating [10,12,35,36]. With reference to the last group—emotion-related variables—as mentioned earlier, COVID-19 can significantly disrupt people’s emotional functioning, and may lead to the experience of severe stress and an increase in eating motivated by the desire to regulate one’s emotional state, which, as is well known from previous studies, can be a very important factor in intensifying emotional overeating [3,5,8,9,11,13,14,37].

Our study aimed to provide an understanding of eating motives and other factors predicting emotional overeating during COVID-19 in a sample of Polish adults. Therefore, in the present study, we examined associations between gender, BMI, eating motives and emotional overeating. Additionally, taking into account the current COVID-19-related situation, we considered the extent to which COVID-19-related stress could be related to emotional overeating. We hypothesised that higher levels of emotion-related predictors (eating motives in the form of affect regulation and COVID-19-related stress) would be associated with higher emotional overeating, after accounting for the effects of demographic variables (gender and BMI) and other eating motives (visual- and attitude-related predictors: liking, pleasure, visual appeal; body- and health-related predictors: need and hunger, health, weight control). This analysis may allow us to determine what factors should be taken into account during COVID-19 in relation to: (a) assessing the risk of the occurrence and intensity of emotional overeating; (b) preventive actions aimed at counteracting emotional overeating; and (c) interventions aimed at reducing emotional overeating. Additionally, it may indicate whether emotion-related predictors are particularly important in predicting overeating; in particular, whether the stress associated with the current pandemic may increase the risk of overeating.

## 2. Materials and Methods

### 2.1. Participants and Procedure

This cross-sectional study was conducted in accordance with the Helsinki Declaration (2001) and was approved by the Ethics Committee (no. 2021/2/2E/2). All adults provided prior written informed consent online and were made aware that participation in our study was voluntary and anonymous.

Participants were recruited via flyers (e.g., online flyers on social media networks, paper flyers posted at universities and various workplaces in the Opole, Silesian and Lower Silesian voivodeships) from January to February 2021 in Poland. Nine hundred and forty-two people volunteered for the study, 74 of whom had significant missing values (data gaps) and were therefore removed from the database. Thus, our final sample included 868 participants (89.06% female) between the ages of 18 and 75 years (*M* = 33.53, *SD* = 11.98). The self-reported body weight ranged from 39 to 146 kg (*M* = 69.18, *SD* = 15.69), height from 146 to 195 cm (*M* = 167.71, *SD* = 7.60) and body mass index from 15.23 to 47.96 kg/m^2^ (*M* = 24.54, *SD* = 5.00). The majority of participants had a healthy body weight (57.60%) and declared their orientation as heterosexual (91.13%) and ethnicity as White (99.65%).

### 2.2. Measures

#### 2.2.1. Eating Motives

The Eating Motivation Survey (brief version) is a 45-item self-report measure of eating motives. It consists of 15 subscales: Liking, Habits, Need & Hunger, Health, Convenience, Pleasure, Traditional Eating, Natural Concerns, Sociability, Price, Visual Appeal, Weight Control, Affect Regulation, Social Norms, Social Image [24]. Participants rate their level of agreement with items on a 7-point Likert-type scale (ranging from “never” to “always”). The higher the score, the more strongly eating is determined by the given motive. In our analysis, we used only some of the subscales. Internal consistency coefficients were acceptable: α_Liking_ = 0.87, α_Pleasure_ = 0.81, α_Visual Appeal_ = 0.81, α_Need & Hunger_ = 0.80, α_Health_ = 0.80, α_Weight Control_ = 0.80, α_Affect Regulation_ = 0.87.

#### 2.2.2. Emotional Overeating

The Emotional Overeating Questionnaire is a 9-item self-reported measure of overeating in response to anxiety, sadness, loneliness, tiredness, anger, happiness, boredom, guilt and physical pain [38]. This questionnaire measures the frequency over the last 28 days. The scores range from 0 (“no days”) to 6 (“every day”). The higher the score, the more emotional overeating over the prior 28 days. In our study, Cronbach’s alpha coefficient was 0.91.

#### 2.2.3. COVID-19-Related Stress

To measure the level of COVID-19-related stress, participants were asked the question “Is the current epidemiological situation related to COVID-19 stressful for you?”. This variable is dummy coded and participants could choose the following responses: “No” (0), “Yes” (1).

#### 2.2.4. Socio-Demographic Variables

Participants were asked about gender, age, weight, height, ethnicity and sexual orientation. We calculated BMI (kg/m^2^) based on self-reported weight and height.

### 2.3. Statistical Analysis

To verify our hypotheses, hierarchical regressions with emotional overeating as a criterion variable were computed. As a first step, we entered demographic predictors (gender and BMI); second step: visual- and attitude-related predictors (liking, pleasure, visual appeal); third step: body- and health-related predictors (need and hunger, health, weight control); and the fourth step: emotion-related predictors (affect regulation, COVID-19-related stress). This allowed us to explore the extent to which affect regulation and COVID-19-related stress incrementally forecasted emotional overeating once the variance related to the other variables had been accounted for. The assumptions for multiple regression analysis were tested. All of them were met. Multicollinearity was measured by variance inflation factors (VIFs) and tolerance. All VIF values did not exceed 10 and tolerance was higher than 0.2 [39,40].

## 3. Results

### 3.1. Descriptive Statistics

Descriptive statistics and correlations between all variables are reported in Table 1.

### 3.2. Regression Analyses

All steps of the regression were significant (Table 2). The first step of the model indicated that female gender and higher BMI were associated with greater emotional overeating. When visual- and attitude-related predictors (liking, pleasure, visual appeal) were added to the model, female gender and BMI remained significant. Moreover, liking and pleasure turned out to be the significant predictors, with the relationship to the first variable being negative and, to the second, positive. Taking into account body- and health-related predictors (need and hunger, health, weight control) the third step resulted in significant predictors: female gender, liking, pleasure and health. The relationship with the newly introduced predictor (health) was negative. In the final model, following the introduction of emotion-related predictors (eating motives in the form of affect regulation and COVID-19-related stress), a positive correlation was observed between emotional overeating and pleasure, affect regulation and COVID-19-related stress, and a negative relationship with visual appeal and health. It was the all-factor model (step 4) that most accurately explained the variance of emotional overeating. Therefore, this means that when planning risk assessment of emotional overeating, preventive actions and interventions, it will be most useful to take into account those predictors that were described in the fourth model and turned out to be significant. This analysis also confirmed that emotion-related predictors were particularly crucial in predicting overeating and, importantly, that COVID-19-related stress could increase the risk of overeating (Table 2).

## 4. Discussion

The results of the present study confirm that emotion-related predictors (eating motives in the form of affect regulation and COVID-19-related stress) are associated with higher emotional overeating, over and above the variance explained by demographic variables (gender and BMI) and other eating motives (visual- and attitude-related predictors: liking, pleasure, visual appeal; body- and health-related predictors: need and hunger, health, weight control). Although, based on our study, we cannot speak to a cause-and-effect relationship, it is possible that COVID-19-related stress leads to reducing the resources used to cope with emotions and stress in everyday life, which in turn leads to overeating [11,12,13,37]. This may be additionally reinforced by the persistence of the tendency to regulate emotions with the use of food (eating motive in the form of affect regulation).

The feeling of COVID-19-related stress can also aggravate preoccupation with and the frequency of negative ruminations about health, the body and eating, and, due to the lockdown, controlling them is much more difficult [18,19,41,42,43]. In the current pandemic, constant health threats and increasing body weight (due to, among other things, limited regular physical activity) can be a source of additional, severe stress, which significantly depletes the resources needed to successfully cope with everyday life [17,18,19,20]. Individuals may wish to gain a sense of control by engaging in unhealthy weight control and/or weight reduction behaviours (e.g., restrictive diets), which in turn can become sources of severe stress and increase the intensity of rumination [42,44]. All of this can lead to the exhaustion of the resources of self-control that we use in everyday life to deal with various situations [45,46]. The described mechanism can lead to the fact that people will take advantage of the “simple” and currently available mechanism of dealing with emotions by overeating. Moreover, overeating may also be associated with an experiential avoidance strategy. As is well known, the unwillingness to remain in contact with aversive experiences and/or taking actions to distract from unpleasant experiences or triggers that cause them can negatively impact our mental health, and can be especially important in the development and maintenance of psychological distress [47].

In the final model, eating motives such as pleasure, visual appeal and health are also significant predictors of emotional overeating. The relationship between the first of these motives and emotional overeating is positive, and negative with the other two.

One of the first studies to investigate the relationship between perceived stress, emotional eating and food choice motives during the COVID-19 pandemic is a study conducted on the American population [48]. According to the findings, emotional eating mediated between stress and food choice motives, such as: mood, sensory attractiveness, comfort and knowledge. The greater the tendency of emotional eating, the greater the desire to choose food based on the abovementioned factors. Our research does not confirm such a relationship in terms of the motive of sensory attractiveness of the food chosen in the context of emotional overeating during the COVID-19 pandemic. The authors of the cited studies explain this state of affairs by the fact that sensory attractiveness, the mere presentation of food as a motivator for emotional overeating, offers a psychological escape and a possible distraction from a stressful situation [49]. Moreover, everything that is aesthetic engages our senses and focuses our attention on experiencing it while distracting our attention from other matters, here, from a pandemic situation [48]. Our results show the opposite and can be explained by the fact that, as suggested by Pope et al. [50], satisfying the desire to eat an attractive product may be replaced by visual satiety. This observation concerns mainly media content (photos, advertisements of dishes), which have become “substitute gluttony” or “substitute consumption” that many people succumb to [50]. Indeed, in the media today, we can see a lot of cooking shows, food advertisements and pictures of food posted by influencers on social media, which can lead to “substitute consumption”. The COVID-19 pandemic has also caused us to spend more time on screen time, whereby the mechanism described above can be stronger [51,52].

It is also worth emphasising here that the motivations related to food choices most often go beyond the nutritional value of food [48]. The findings of other researchers indicate that the “willingness to be healthy” is often associated with a healthy lifestyle, which is most often manifested by the strength of a person’s motivation to eat a healthy diet [53,54]. Interestingly, during the COVID-19 pandemic, significant changes in the motives of food choice have already been observed in the context of the increased importance of these choices for health [55], which is confirmed by our results. Data on the nutritional motivation of Irish adults also corresponds to our results, with people with strong motivation to follow a healthy diet having a healthier nutritional profile [53]. This may mean that some people will find healthy eating an important factor in maintaining health, which is the focus of many of us today.

Our study also found an increase in emotional overeating due to pleasure motivation. Given that eating is one of the main pathways to pleasure [32], our results confirm this. We read in the literature that the possibility of enjoying food also triggers other pleasant experiences (e.g., sense of security, relaxation) and we can observe that various brain mechanisms are responsible for the relationship between eating and feeling pleasure [32]. Therefore, it should be considered whether food may be an important source of pleasure during a pandemic in which many ways of achieving pleasure have been reduced.

To sum up, during the COVID-19 pandemic, eating can, on the one hand, help to cope with the current difficult situation and the negative emotions associated with it; on the other hand, frequent use of this tendency can lead to rigid regulation of affect and use of this mechanism as the dominant mechanism [11,12,13,37,56]. Therefore, limited social contact, related disruptions in daily activities and stress resulting from COVID-19 should generate appropriate interventions, not necessarily focusing only on emotional eating, but also on the resources of the individual. It is worth encouraging specialists to implement alternative methods of contact with their patients, e.g., online.

It is important to conduct follow-up surveys to shed more light on emotional overeating, demographic variables, eating motives and COVID-19-related stress. Future studies could include, inter alia, analysis of: (1) data according to age range (e.g., young people 18 to 30 years old, adults 30 to 55 years old, people over 55 years old); (2) other important variables: lifestyle, relationship status (who the person lives with), employment status, anxiety and depression; (3) more detailed information on the COVID-19 situation (whether participants or their relatives have had COVID-19; whether any of the participants’ relatives have died from COVID-19); (4) other eating motives; (5) the interaction between COVID-19-related stress and other variables, e.g., affect regulation, gender (these analyses would explain why COVID stress and/or affect regulation are mediating their direct effects).

The present study has certain limitations. The main ones are: (a) this study relies on a cross-sectional design which limits the possibility of drawing conclusions about cause–effect relationships; (b) the measurement of variables is based on self-report; (c) we used one item to measure COVID-19-related stress; (d) a convenience sampling method was used to recruit participants. It is absolutely necessary to take these limitations into account when planning further studies, especially because the latest literature provides certain solutions in this regard (e.g., measurement of stress related to COVID-19 [57]). Finally, it should be emphasised that, taking into account the above limitations and the specificity of the lockdown conditions in Poland, our results may have limited generalisability.

## Figures and Tables

**Table 1:** Descriptive statistics and correlations of study variables for the whole group.

	1	2	3	4	5	6	7	8	9	10	11
1. Gender ^1^											
2. BMI	−0.10 **										
3. Liking	−0.01	−0.08 *									
4. Pleasure	−0.002	−0.02	0.66 ***								
5. Visual Appeal	0.02	−0.03	0.45 ***	0.61 ***							
6. Need and Hunger	−0.01	−0.10 **	0.72 ***	0.64 ***	0.50 ***						
7. Health	0.03	−0.14 ***	0.43 ***	0.35 ***	0.31 ***	0.61 ***					
8. Weight Control	0.07 *	0.01	0.28 ***	0.35 ***	0.37 ***	0.43 ***	0.67 ***				
9. Affect Regulation	0.09 **	0.10 **	0.30 ***	0.61 ***	0.50 ***	0.35 ***	−0.17 ***	0.35 ***			
10. COVID-19-Related Stress ^1^	0.18 ***	0.01	−0.01	0.09 **	0.06	0.003	−0.02	0.06	0.17 ***		
11. Emotional Overeating	0.08 *	0.08 *	0.02	0.22 ***	0.10 **	0.02	0.10 **	0.02	0.44 ***	0.15 ***	
*M*		24.54	14.82	11.02	9.23	12.52	11.64	9.98	7.83		6.23
*SD*		5.00	4.13	4.22	3.94	3.96	4.42	4.29	4.42		8.18

^1^ Dummy-coded variables (gender: 0—men, 1—women; COVID-19-related stress: 0—no, 1—yes); BMI—body mass index; * *p* < 0.05, ** *p* < 0.01, *** *p* < 0.001.

**Table 2:** Results of multiple hierarchical regression analyses for the prediction of emotional overeating.

		Emotional Overeating				
Step	Variables	B	SE	*β*	*t*	*p*
1		*F*(2, 867) = 6.05, *p* < 0.01, Adj. *R*^2^ = 0.01				
	Gender	2.21	0.89	0.09	2.50	0.013
	BMI	0.15	0.06	0.09	2.65	0.008
2		*F*(5, 867) = 16.92, *p* < 0.001, Adj. *R*^2^ = 0.08 (Δ*F p* < 0.001)				
	Gender	2.19	0.85	0.08	2.57	0.010
	BMI	0.13	0.05	0.08	2.43	0.015
	Liking	−0.40	0.09	−0.20	−4.65	<0.001
	Pleasure	0.74	0.09	0.38	7.87	<0.001
	Visual Appeal	−0.09	0.09	−0.04	−1.04	0.300
3		*F*(8, 867) = 13.46, *p* < 0.001, Adj. *R*^2^ = 0.11 (Δ*F p* < 0.001)				
	Gender	2.20	0.85	0.08	2.60	0.010
	BMI	0.09	0.05	0.06	1.70	0.090
	Liking	−0.27	0.10	−0.14	−2.68	0.008
	Pleasure	0.76	0.10	0.39	7.86	<0.001
	Visual Appeal	−0.07	0.09	−0.03	−0.70	0.429
	Need and Hunger	−0.03	0.12	−0.02	−0.26	0.793
	Health	−0.36	0.09	−0.19	−3.84	<0.001
	Weight Control	0.11	0.09	0.06	1.29	0.199
4		*F*(10, 867) = 27.20, *p* < 0.001, Adj. *R*^2^ = 0.23 (Δ*F p* < 0.001)				
	Gender	0.81	0.80	0.03	1.01	0.315
	BMI	0.02	0.05	0.01	0.44	0.661
	Liking	−0.12	0.09	−0.06	−1.25	0.213
	Pleasure	0.21	0.10	0.11	2.06	0.040
	Visual Appeal	−0.25	0.08	−0.12	−2.99	0.003
	Need and Hunger	−0.05	0.11	−0.02	−0.45	0.652
	Health	−0.19	0.09	−0.10	−2.15	0.032
	Weight Control	−0.11	0.08	−0.06	−1.29	0.196
	Affect Regulation	0.88	0.08	0.48	11.63	<0.001
	COVID−19−Related Stress	1.14	0.53	0.07	2.17	0.031

BMI—body mass index.

## Data Availability

The data that support the findings of this study are available from the corresponding author upon reasonable request.
